# Development and Beam-Shape Analysis of an Integrated Fiber-Optic Confocal Probe for High-Precision Central Thickness Measurement of Small-Radius Lenses

**DOI:** 10.3390/s150408512

**Published:** 2015-04-13

**Authors:** Boonsong Sutapun, Armote Somboonkaew, Ratthasart Amarit, Sataporn Chanhorm

**Affiliations:** 1 School of Electronic Engineering, Institute of Engineering, Suranaree University of Technology, 111 University Ave., Muang, Nakhon Ratchasima 30000, Thailand; 2 Photonics Technology Laboratory, National Electronics and Computer Technology Center (NECTEC), National Science and Technology Development Agency (NSTDA), 112 Thailand Science Park, Pahol Yothin Rd., Klong Luang, Pathumthani 12120, Thailand; E-Mails: armote.somboonkaew@nectec.or.th (A.S.); ratthasart.amarit@nectec.or.th (R.A.); sataporn.chanhorm@nectec.or.th (S.C.)

**Keywords:** fiber-optic confocal microscopy, non-contact thickness measurement, ball lenses, optical metrology

## Abstract

This work describes a new design of a fiber-optic confocal probe suitable for measuring the central thicknesses of small-radius optical lenses or similar objects. The proposed confocal probe utilizes an integrated camera that functions as a shape-encoded position-sensing device. The confocal signal for thickness measurement and beam-shape data for off-axis measurement can be simultaneously acquired using the proposed probe. Placing the probe’s focal point off-center relative to a sample’s vertex produces a non-circular image at the camera’s image plane that closely resembles an ellipse for small displacements. We were able to precisely position the confocal probe’s focal point relative to the vertex point of a ball lens with a radius of 2.5 mm, with a lateral resolution of 1.2 µm. The reflected beam shape based on partial blocking by an aperture was analyzed and verified experimentally. The proposed confocal probe offers a low-cost, high-precision technique, an alternative to a high-cost three-dimensional surface profiler, for tight quality control of small optical lenses during the manufacturing process.

## 1. Introduction

Optical devices and instruments have become smaller and currently require very compact and high-quality optical lenses. Manufacturing and assembling such high-quality lenses requires high-tolerance quality control for every single lens. One parameter often measured and monitored during the production process is the central thickness of a lens. A precise, non-contact measuring system based on an optical technique is preferred for lens-thickness measurement owing to its high sensitivity, minimal invasiveness, and rapid measurement.

Non-contact techniques that have been widely employed for thickness measurement of transparent plates and optical lenses are mostly based on low-coherence interferometry [1,2,3,4,5,6,7,8] and confocal microscopy [9,10,11,12,13]. Interferometry-based techniques can be used to measure thicknesses of transparent objects in the range of ten microns to a few millimeters, with sub-nanometer resolution [1,2,4,5]. However, their high cost and high sensitivity to vibrations, temperature drift, and air turbulence make interferometric methods rather unsuitable for routine usage on the factory floor [14]. Confocal microscopy, in particular fiber-optic microscopy, is less subject to environmental disturbances, owing to its ability to reject out-of-focus signals and the narrow full-width-at-half-maximum (FWHM) of the confocal peak—As small as a few microns—Which is therefore detected only when the focal point is precisely positioned on a sample’s surface [15,16]. Fiber-optic microscopy has other advantages, including relatively little complexity, simple optical alignment, suitability for most types of materials, high precision, and smaller probe size. A fiber-optic confocal microscope requires an axial scanning mechanism to move either the probe or the sample so that the probe’s focal point is positioned along the sample surfaces; such scanning mechanisms may be classified into two groups: mechanical axial scanning [10,15,16,17] and chromatic depth scanning [18,19,20,21,22]. Commercial fiber-optic confocal microscopes have gained increasing acceptance among manufacturing industries. Such devices can have a measurement range of several millimeters with a depth resolution as small as a few nanometers [23].

There are reports of using a reflection confocal sensor to measure the central thickness of a lens [9,10,11,24]. During depth scanning, the optical distance is calculated from the interval between confocal peaks corresponding to the top and bottom surfaces of the lens. To determine the central thickness of the lens from the optical distance, the radius of curvature of the upper surface and refractive index of the lens as well as the numerical aperture (NA) of the confocal probe must be taken into account [9,11]. It is also possible to determine both the thickness and refractive index of a lens from the confocal signal using a ray-tracing iteration technique [10].

It is important to point out that for central thickness measurement of small-diameter optical lenses, the instrument resolution is not the only parameter that affects measurement accuracy; the ability to precisely place the instrument’s sensing spot at the vertex of a measured lens is also crucial. A slightly off-center measurement (e.g., less than the centering accuracy of a device) results in a large error in the thickness value, particularly for millimeter-size lenses. A general approach for solving this problem is to install a single-point instrument on a precision translation stage and utilize point-to-point scanning in the *xy*-plane. This technique, however, is relatively slow, provides no feedback and may experience an instrument drift error. Another solution to the problem is to utilize a 3D surface profile instrument to perform a surface profile measurement [13,16,19,25]. However, the high cost and complexity of such an instrument may make this technique impractical for routine production processes.

We have previously proposed a new design for a fiber-optic confocal probe that can simultaneously measure the thickness and locate the position of the vertex point of a lens sample relative to the focal point of the probe [26]. The key design innovation of that proposed confocal probe was the addition of a camera and a beam splitter to the confocal probe, allowing the shape of the reflected light beam to be detected. In this work, we further provide theoretical analysis of the integrated confocal probe.

We have chosen glass ball lenses with radii between 1.5 mm and 5.0 mm as examples of small-radius lenses. Nevertheless, our proposed technique can be applied to any type of lens, as well as to other materials. We have demonstrated that the modified confocal probe can be used effectively to measure the displacement between the focal point of the probe and the vertex point of a ball lens, even if they are only a few microns apart. As a result, the central thickness of the ball lens can be measured with high precision using the same probe, but in a confocal mode. In practice, the shape of the reflected beam, which can be monitored in real time during the measurement, may serve as a warning signal or a feedback if the off-center displacement is out of the preset range. In this report, shape analysis of the reflected beam based on geometrical optics is described in detail. Calculated results are verified and compared with experimental results.

## 2. Device Sensing Principles

### 2.1. Confocal Probe Design

**Figure 1:**
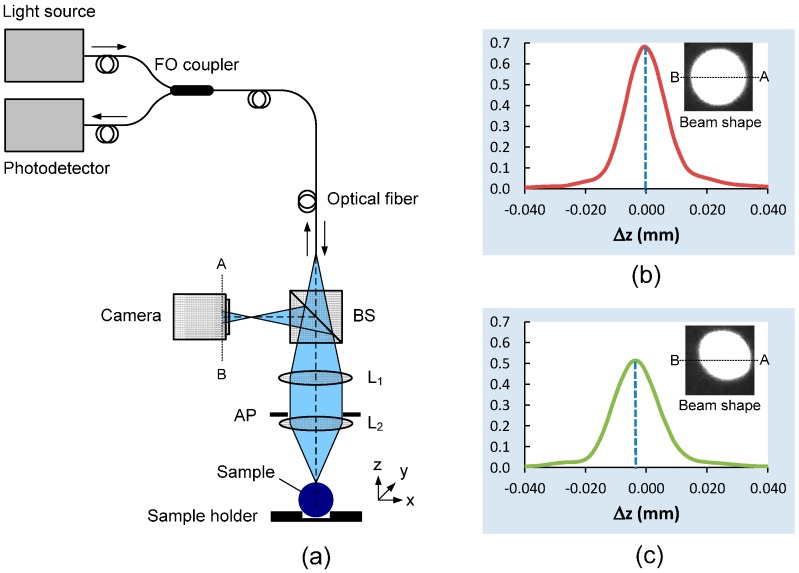
(**a**) Schematic of the integrated fiber-optic confocal probe. A camera integrated with a confocal probe acts as a vertex sensing device. BS—beam splitter, AP—aperture, L_1_—achromatic lens and L_2_—10× microscope objective; (**b**) A confocal signal measured by the photodetector when the probe’s focal point is exactly at the lens vertex. The inserted image is the corresponding beam shape; (**c**) A confocal signal and the corresponding beam shape when the probe’s focal point is slightly displaced from the vertex.

Figure 1 shows a schematic of the proposed confocal probe for a scanning confocal microscope. The fiber-optic confocal probe used in this work has a similar design to the probes reported in previous works [9,11,12,13,15,27], except that a camera is added to the probe to serve as a shape-encoded position-sensing device. A laser diode or a superluminescent diode (SLD) light source is coupled to the input port and its light emerges from the output ports of a 1 × 2 single-mode fiber-optic coupler. An optical fiber with a small core dimension serves simultaneously as a point light source and a point receiver for back-reflected light. The output light beam is collimated by a collimating lens L_1_ and then focused to a spot by an objective lens L_2_. Light reflected from the sample surface travels back toward the confocal probe, into the fiber-optic coupler and then to a photodetector that measures the light intensity. Only when the focal spot of the focusing lens L_2_ is positioned exactly at the sample surface is the reflected light focused back to the endface of the optical fiber by lens L_1_ and coupled efficiently into the optical fiber, and thereby the light intensity measured by the photodetector is highest. If the focal spot is positioned above or below the sample surface, the reflected light is broadened at the fiber’s endface and coupling efficiently into the fiber will be low. By scanning the sample or the probe focal point axially, a confocal response curve can be obtained and the peak position that corresponds to the position of the sample surface can then be calculated.

In our probe design, a beam splitter (BS) was added to the optical path of the probe to partially deflect the reflected light to a camera, which functions as a vertex sensor. The camera is positioned a certain distance behind the focal point of the reflected light to allow the shape of the light beam to be clearly observed. When the focal point of the confocal probe is exactly at the vertex of the ball lens sample, the beam shape of the reflected light at the camera is a circular spot (Figure 1b). Also, the confocal signal measured by the photodetector is highest at this position. When the focal point of the confocal probe is moved away from the lens vertex in transverse direction but still focused on the sample’s surface, the beam shape is non-circular and resembles an ellipse (Figure 1c). This is due to the sample plane being tilted relative to the probe optical axis and a certain segment of a reflected light beam being blocked by the aperture (AP) and not detected by the camera. The larger the off-center displacement, the greater is the tilted angle and the distortion of the beam shape. The confocal intensity is lower than that measured at the vertex owing to the same reason. Also, the confocal peaks in Figure 1b,c are at different axial positions. It is apparent that measurement of the confocal peak at this off-center position will cause an error to the central thickness measurement.

### 2.2. Experimental and Calibration

The confocal probe setup was built based on the proposed device shown in Figure 1. Light output from an 850-nm SLD (3.75 mW, 50-nm bandwidth, EXALOS, Schlieren, Switzerland) was coupled to the input port and emerged at the output ports of a 1 × 2 50:50 single-mode fiber-optic coupler. A laser source with a suitable speckle reducer may replace the SLD to obtain a beam image with low speckle noise. The confocal probe was composed of a collimating lens L_1_ and a focusing lens L_2_. L_1_ is an achromatic doublet (f = 100 mm, Thorlabs, Newton, NJ, USA) and L_2_ is a 10× microscope objective (#RMS10X, infinite-corrected, NA = 0.25, Olympus, Waltham, MA, USA). The ball lens sample was scanned using an xyz linear stage. The z-axis stage was a motorized linear stage (#MFA-CC, 0.017-µm resolution, Newport, Irvine, CA, USA), while the *x* and *y* stages were manual stages. The axial confocal response curve was obtained by scanning the sample along the axial axis (*z*-axis) and recording the light intensity at the photodetector. The confocal system has an axial resolution of 0.39 µm.

A beam splitter BS partially deflected the reflected light to a camera (#scA1400-17gm, pixel size: 6.45 × 6.45 µm, Basler, Ahrensburg, Germany). The light intensity signal from the photodiode, the position from a *z*-axis linear stage, and the images from the camera were acquired and analyzed by a computer using a custom program developed with LabVIEW software (National Instruments, Austin, TX, USA).

**Figure 2:**
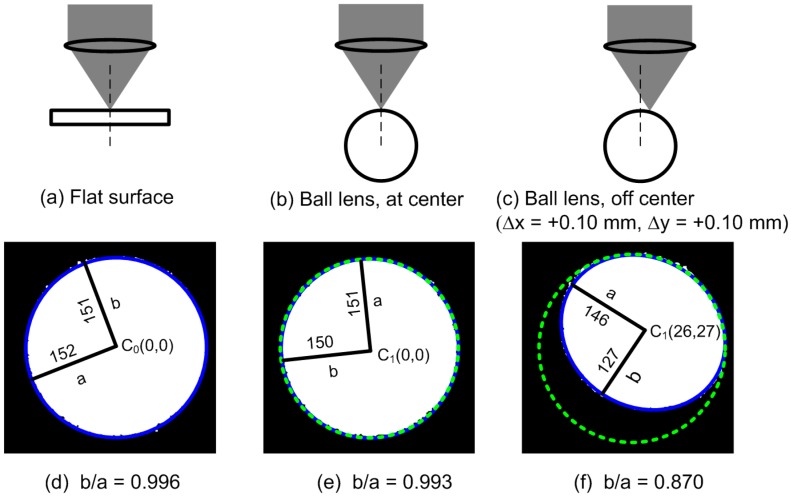
Calibration and characterization of the beam shape. (**a**) The confocal probe’s focal point was positioned on the top surface of a flat glass plate; (**b**) The probe’s focal point was located at the sample vertex of a 2.5-mm-radius ball lens; (**c**) The probe’s focal point was focused on the top surface but displaced from the sample vertex by ∆*x* = +0.10 mm and ∆*y* = +0.10 mm; (**d**–**f**) are the beam images for (**a**–**c**), respectively. The green dotted circle shown in (**e**,**f**) is the reference circle obtained from the beam image in (**d**) (blue solid circle) and redrawn for comparison. *C*_0_ is the center of the reference image, *C*_1_ is the center of the measured image, *a* is the semi-major axis, and *b* is the semi-minor axis of the fitted ellipse. Note that all ellipse parameters are given in units of camera pixels.

To calibrate the measurement setup, the ball lens sample was replaced with a flat glass plate (Figure 2). The flat glass plate was manually adjusted until the top surface was at the focal point of the confocal probe and the sample plane was perpendicular to the optical axis of the probe. At this position, the confocal signal was strongest and the beam image at the camera appeared as a circular spot.

The center and the diameter of the recorded image were fitted using an ellipse function available in the LabVIEW software. The perimeter of the recorded image and its center position were set as the reference circle and as the reference center of the ellipse *C*_0_(0, 0, in units of pixels) (see Figure 2d), respectively, for subsequent experiments. Note that when the sample was moved out of this position along the *z*-axis, the image remained a circular spot, but became blurred. The intensity of the beam image also dropped rapidly.

Three glass ball lens samples (Edmund Optics, Barrington, NJ, USA) with radii 1.5, 2.5 and 5.0 mm were used in this experiment. We first adjusted the *xy* and *z* positions of a lens sample so that its vertex was at the beam’s focal point by simply monitoring the beam shape until it resembled the reference circle. We then moved the lens sample to various slightly off-center positions (∆*x*, ∆*y*) in the *xy*-plane using the manual linear stages, and then performed an axial scan. We then captured the corresponding reflected beam images at the peak position of the confocal signals. The reflected beam shapes were apparently ellipses and were well matched to ellipse parameters obtained using the ellipse function in the LabVIEW software.

We therefore envisioned using ellipse parameters obtained from a beam image to determine the position of the probe’s focal point relative to the lens vertex.

To verify that we can use the ellipse parameters of the reflected beam image to determine the position of the probe’s focal point, we first determined the best-fitted ellipse for the recorded beam shape and then calculated the semi-major axis *a*, the semi-minor axis *b* and the center of the ellipse *C*_1_(*x*, *y*) pixels (see Figure 2e,f). We then defined the ellipse axis ratio as *b/a*. Note that the ellipse axis ratio is one for a perfectly circular image and always less than one for an ellipse. For each ball lens, the ellipse axis ratios *b/a* were determined and plotted as a function of off-center displacements (∆*x*, ∆*y*) relative to the lens vertex.

## 3. Results and Discussion

**Figure 3:**
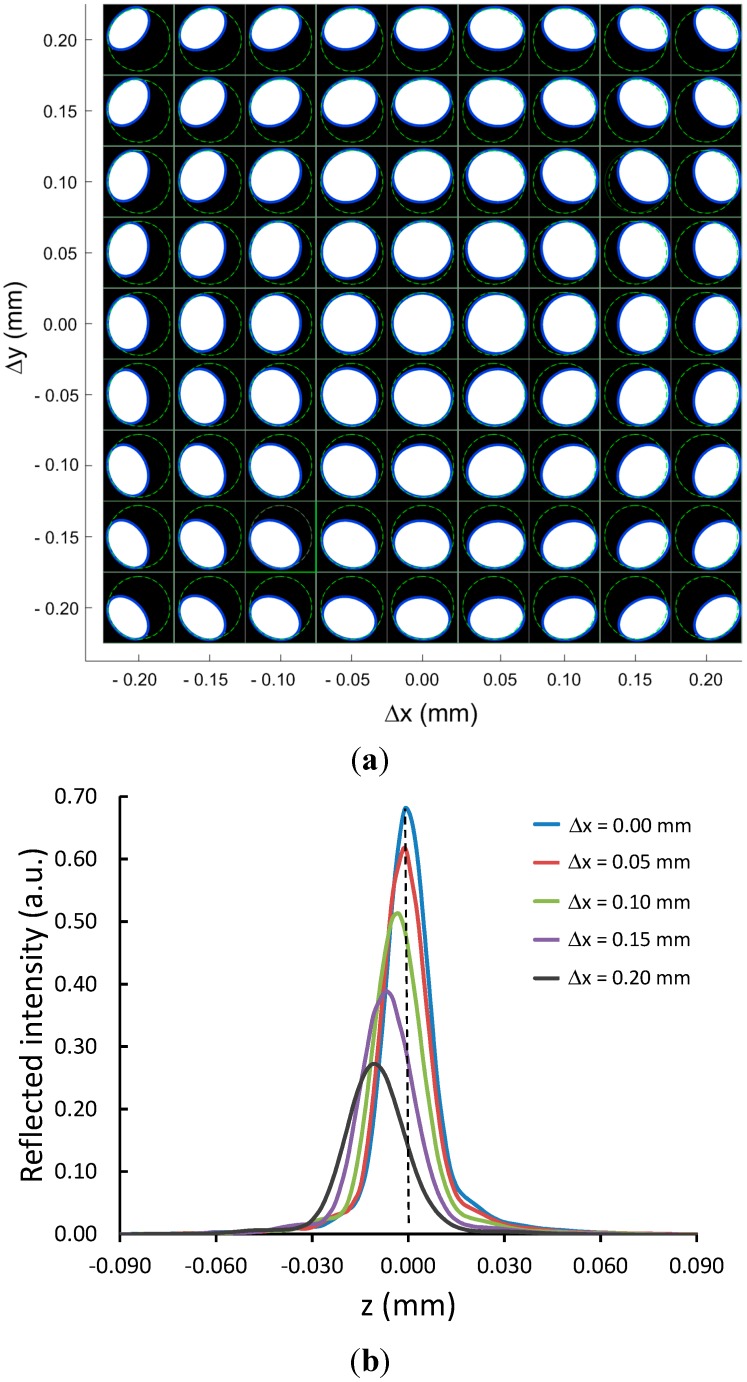
(**a**) Shapes of the reflected light beam when the confocal probe was focused onto the top surface of a 2.5-mm-radius ball lens at various off-center displacements (*∆x*, *∆y*) from the sample vertex. An off-center displacement of the probe focal point relative to the lens vertex produced a non-circular image. The reflected beam images were well fitted as an ellipse (**blue solid circle**). The reference circle obtained from the flat glass plate is also given (**dashed green circle**). The image frame size of each image is 360 × 360 camera pixels; (**b**) The corresponding confocal signals along the +*x*-axis for *∆x* = 0.00, 0.05, 0.10, 0.15 and 0.20 mm.

Figure 3a shows the images of the reflected beam measured at the camera plane when the focal point of the confocal probe was on the top surface of a 2.5-mm-radius ball lens at various displacements (∆*x*, ∆*y*) around the vertex. When the focal point of the probe was exactly at the lens vertex (the image at the middle of Figure 3a), the beam shape was circular with a calculated ellipse axis ratio of 0.992, which is close to 1. It can be seen from the figure that moving the lens sample’s vertex slightly away from the probe’s focal point along the *xy*-plane produced a non-circular beam spot in which a certain segment of the circle was removed from the reference image. These images appeared to be well fitted as ellipses when the off-axis displacement was within a ±0.20 mm range from the vertex. For a larger off-center displacement, we found that the beam image was severely distorted compared to a reference circle and was therefore not well fitted to an ellipse.

Figure 3b shows the corresponding confocal signals for the images in Figure 3a along the +*x*-direction for *∆x* = 0, 0.05, 0.10, 0.15 and 0.20 mm. The confocal signal obtained at the vertex (*∆x* = 0 mm) showed the highest intensity and its peak position was intentionally set to *z* = 0.000 mm. The peak positions of the confocal signal dropped below the *z*-value at the vertex if the probe focal point was not at the vertex. For example, when the probe was displaced by *∆x* = 0.10 mm, the confocal peak position was approximately 4 μm below the *z*-value found at the vertex.

**Figure 4:**
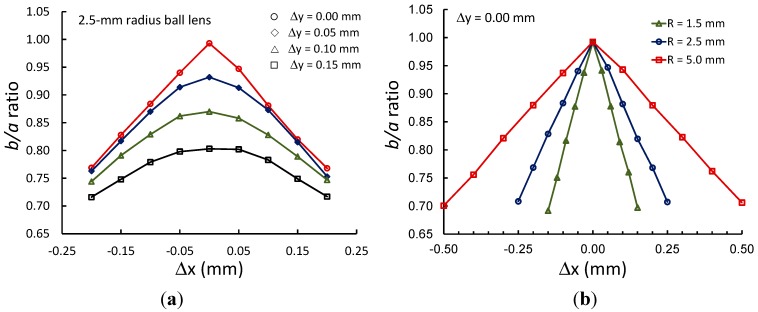
(**a**) The ellipse axis ratios *b*/*a* plotted as a function of the probe’s focal point displacement from the lens vertex on the *x*-axis (the beam images in Figure 3a for *∆y* = 0.00, 0.05, 0.10 and 0.15 mm) for a 2.5-mm-radius ball lens; (**b**) Axis ratio curves for three different ball lenses with radii of 1.5, 2.5 and 5.0 mm. Note that the centers of the ball lenses were intentionally set at *C*_0_(0, 0) pixels.

Therefore, a small off-center displacement would cause a large error in the central thickness measurement. The confocal signals were broadened and their peak intensities also dropped sharply as we moved the probe’s focal point further in the *x* direction away from the vertex, since the sample plane was now significantly tilted relative to the probe’s optical axis. Figure 4a shows the elliptical axis *b*/*a* ratios obtained from the recorded images in Figure 3a, plotted as a function of the off-center displacement along the *x*-axis for different values of *∆y* in the range of 0.00–0.15 mm. There was a linear relationship between the ellipse axis ratio and the displacement for *∆y* = 0 mm (*x*-axis) but not for other values of *∆y*. Similar results were obtained when we plotted the ellipse axis ratios as functions of the off-center displacement along the *y*-axis. Using the data obtained from Figure 3a, we found that a surface plot of the ellipse axis ratio as a function of an off-axis displacement was a cone shape with the maximum value at zero displacement. This ratio decreased more rapidly for a ball lens with a smaller diameter, as shown in Figure 4b.

The beam images presented in Figure 3a and Figure 4 demonstrate that the shape of a reflected light beam not only depends on the off-center displacement but is also directionally dependent. For example, when we moved the probe focal point away from the vertex along the +45°-line with respective to the +x-axis (the ball lens sample was moved in the opposite direction, −135° with respective to the +x-axis), the removed segment of a circular beam image was in the third quadrant. On the other hand, when we moved the probe’s focal point away from the vertex along the −135° line with respect to the +*x*-direction, the removed segment of a circular beam image was in the first quadrant.

In addition, we observed that the ellipse center, *C*_1_(*x*, *y*), of the beam image moved away from the reference origin *C*_0_(0, 0) in the same direction that the focal point moved from the vertex. As an example, considering the beam images in Figure 3a, the ellipse center was at *C*_1_(25, 28) pixels for the focal point displacement of *∆x* = +0.10 mm and *∆y* = +0.10 mm from the lens vertex. In the opposite direction, the ellipse center was at *C*_1_(−26, −28) pixels for the focal point displacement of *∆x* = −0.10 mm and *∆y* = −0.10 mm from the lens vertex. Therefore, one may use the elliptical axis ratio data as well as the center of the ellipse as a simple visual guideline or as a feedback signal to aid in alignment of the focal point of the probe to the vertex of the lens sample.

**Figure 5:**
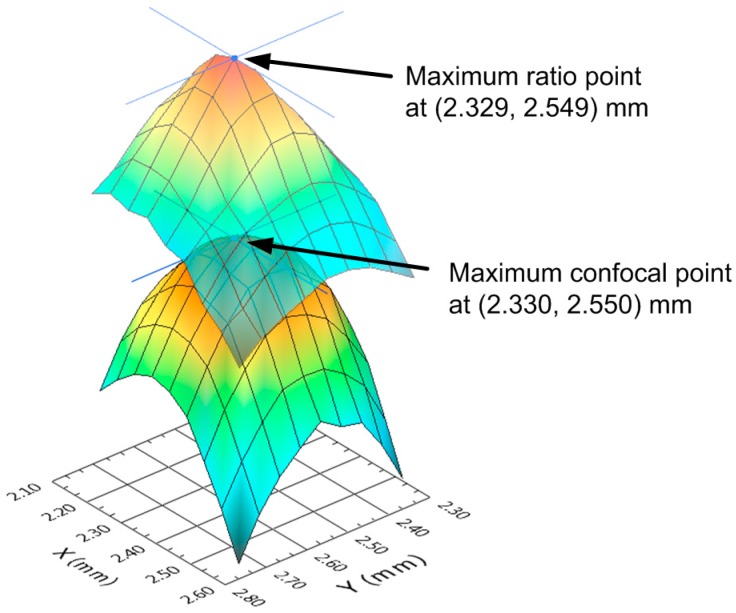
Ellipse axis ratio profile (**Top**) and lens surface profile (**Bottom**) for a 2.5-mm-radius ball lens sample. The apex points calculated from the axis ratio and the surface profile plots are (*x*, *y*) = (2.329, 2.549) mm and (*x*, *y*) = (2.330, 2.550) mm, respectively. Both apex points are at nearly the same position.

To further verify that the position at which the reflected beam reaches its maximum ellipse axis ratio is physically at the vertex point of the ball lens, we scanned the 2.5-mm-radius ball lens in 10-µm steps along the *xy*-plane over an area of 0.50 × 0.50 mm around the vertex point and measured both the confocal peak position and the ellipse axis ratio. Figure 5 shows the ellipse axis ratio profile plotted along with the confocal peak position profile (*i.e.*, the lens surface profile). Using a centroid calculation technique, we determined that the ellipse axis ratio was highest (*b*/*a* = 0.997) at (*x*, *y*) = (2.329, 2.549) mm, while the confocal peak position was highest at (*x*, *y*) = (2.330, 2.550) mm. These two points were calculated to be only 1.4 µm apart. These results indicate that both points were at nearly the same position.

**Figure 6:**
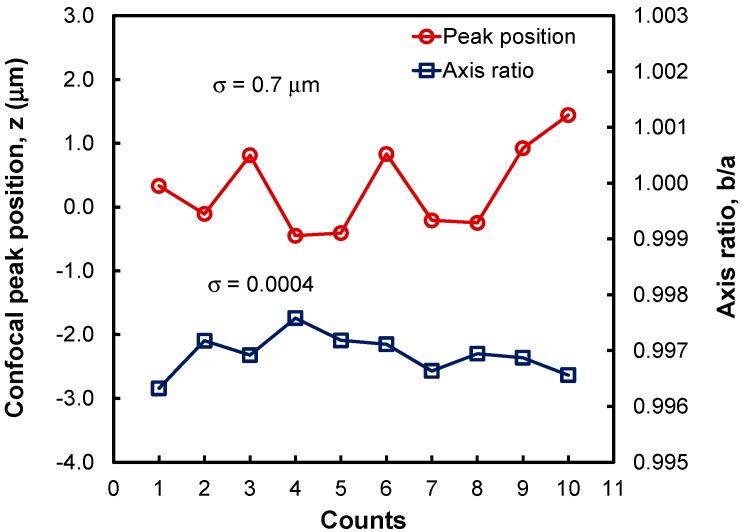
Confocal peak position and ellipse axis ratio values obtained in 10 successive measurements of a 2.5-mm-radius ball lens sample. For each measurement, the ball lens sample was removed and then reinserted into the sample holder followed by a focal point-lens vertex alignment using the beam-shape image data. The standard deviations of the peak position detection and the ellipse axis ratio measurement were 0.7 μm and 0.0004, respectively.

Figure 6 shows the ellipse axis ratios and the confocal peak positions of 10 successive measurements using the 2.5-mm-radius ball lens sample. The sample was reinserted into the sample holder before each measurement. We used both the image shape and the ellipse axis ratio as a guideline to realign the probe’s focal point to the sample’s vertex. We found that it was quick and simple to realign the lens vertex to the probe’s focal point, because the beam shape and the ellipse axis ratio could be monitored in real time.

From the data in Figure 4 and Figure 6, the measured device sensitivity—Defined as how much the ellipse axis ratio changes as a function of off-center displacement—And the standard deviation (σ) for the ellipse axis ratio were 0.97 mm^−1^ and 0.0004, respectively, for the 2.5-mm-radius lens. We then calculated the lateral resolution of this system as 3 × (0.0004)/(0.97) = 1.2 µm. For a 2.5-mm-radius ball lens, the measurement of the confocal peak position has a standard deviation of 0.7 µm. Finally, this device has a resolution for central thickness measurement of lenses = 3 × 0.7 µm = 2.1 µm. Therefore, by simply adding a camera and a beam splitter to a confocal probe, we devised a simple and precise technique suitable for measuring the central thickness of millimeter-size ball lenses and other small optical elements.

The calculated lateral resolution of this system of 1.2 µm for a 2.5-mm-radius ball lens as previously described is in good agreement with the results presented Figure 5 in which we showed that the maximum ellipse ratio and the lens vertex obtained from the lens surface profile for the same ball lens were physically only 1.4 µm apart. These results demonstrate that the proposed device can be used to precisely place the confocal probe’s focal point at the vertex point of a lens even for small radius lenses, and thus it will improve the device’s thickness measurement error.

The position of the confocal probe’s focal point relative to the lens vertex has not been received any attention in the previous works of using a confocal probe to measure the central thickness of a lens [9,10,11,24]. It is probably because of large lens samples being used in the previous reports; therefore, a slightly off-center measurement results in an insignificant error in the thickness measurement value. For example, an off-center displacement of 0.10 mm would have added a negligible error of 0.05 µm to a thickness measurement error of 1.5–2.6 µm for a 100-mm-radius lens sample in [10,24]. In comparison, the same off-center displacement caused a measured thickness error of about 4 µm for a 2.5-mm-radius ball lens.

The confocal probe with an integrated camera described in this work requires an axial scanning method. The technique described (using narrow band width light) cannot be applied to chromatic confocal microscopy without some modification. For chromatic confocal microscopy, different wavelengths of the light source are focused at different depths. Only light with the wavelength whose corresponding focal point is exactly at the sample surface reflects back to and is collected efficiently by the optical fiber and then transmitted to the spectral detection unit. However, light that reflects back to the camera shown in Figure 1a comprises several wavelengths including the dominant wavelength. In order to get a clear beam image of the dominant wavelength, an appropriate variable optical filtering component should be placed in front of the camera. This solution is currently under investigation.

## 4. Shape Analysis of the Reflected Light Beam

**Figure 7:**
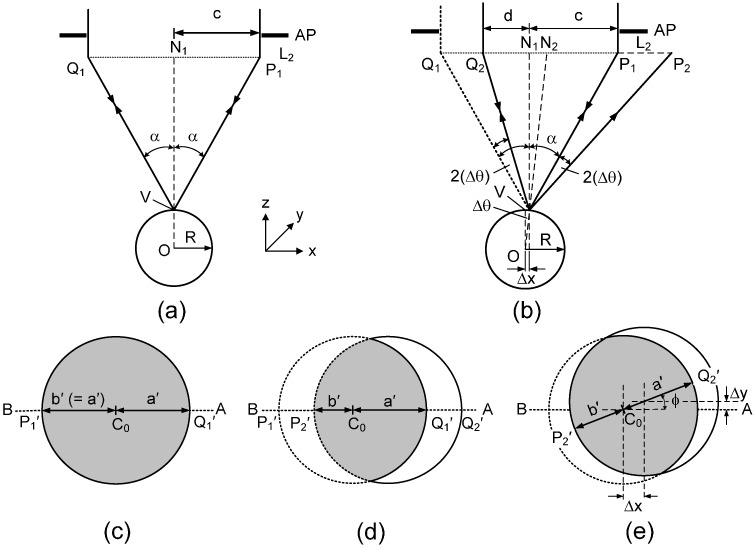
Analysis of the beam shapes at the camera plane (**a**) The focal point of the incident beam is exactly at the vertex of the ball lens sample; (**b**) The ball lens sample is moved in the −*x* direction by a displacement of *∆x*; (**c**,**d**) are the corresponding images at the camera image plane of the reflected beams in the case of (**a**,**b**), respectively; (**e**) The image of the reflected beam when the focal point is displaced by an arbitrary distance *r*(*∆x*, *∆y*).

To fully understand the sensing principles of this device, we provide detailed analysis of the shape of the reflected beam as a function of off-center displacement. As shown in Figure 7, we consider the incident light beam’s passes through the aperture (AP) with a radius of *c* and then is focused by the objective lens L_2_ having a NA = sin (α), where α is the half-aperture angle. When the focal point of the light beam is at the vertex of the lens sample (Figure 7a), the optical axis of the probe coincides with the surface normal (ON_1_) and is also perpendicular to the tangent plane at the vertex point. Rays Q_1_V and P_1_V are reflected back to the objective lens L_2_ and the aperture AP, and then reflected by the beam splitter BS to the camera (see Figure 1a). The beam image of the reflected beam measured at the camera image plane in this case is, therefore, a circular shape with some demagnification (Figure 7c).

In Figure 7b, we assume that the lens sample is moved slightly in the –*x* direction by a distance of *∆x* from its original position in Figure 7a. Therefore, the probe’s focal point is oppositely displaced by a distance of *∆x* in the +*x* direction compared to the sample’s vertex. We assume here that *∆x* << *R*, where *R* is the radius of curvature of the lens sample. The surface normal at the focal point is now along ON_2_. The angle *∆*θ between ON_1_ and ON_2_ can be determined from the displacement *∆x* and the radius of curvature of the lens sample using the following expression:

(1) $$∆\text{θ }=\;\tan^{-1}\left(\frac{∆x}{\sqrt{R^{2}-∆x^{2}}}\right)\;≅\;\frac{∆x}{R}\;;\;∆x\;\ll \;R$$

For this condition, light ray Q_1_V reflects as ray VP_2_ with a reflection angle of α + θ with respect to the normal line ON_2_. This reflected ray VP_2_ is blocked by the aperture AP and will not enter the optical system. On the other hand, ray P_1_V reflects as ray VQ_2_, which is still within the aperture angle of the objective and thus passes through the optical system. The light intensity pattern at the camera image plane is now a distorted circular shape (the gray area in Figure 7d) that has a segment of the original circle removed to form its shape. Here, we define *a'* as the original radius of the circular image and *b'* as the distance measured along the x-axis from the image center (*C*_0_) to the distorted edge. From Figure 7d we see that the ratio *b'*/*a'* of the beam image at the camera plane is equal to the ratio *d*/*c* of the reflected beam at the aperture AP plane. We then determine the distorted beam’s axis ratio, *b'*/*a'* of the image measured at the camera plane, as:

(2) $$\frac{b’}{a’}\;=\;\frac{d}{c}\;=\;\frac{\tan(\text{α}-2∆\text{θ})}{\tan(\text{α})}$$

where *c* and *d* are the original beam radius and the distorted beam radius at the aperture plane, respectively. For small displacements from the lens vertex, using the value of ∆θ from Equation (1), we may estimate the distorted beam ratio:

(3) $$\frac{b’}{a’}\;≅\;1-\frac{2}{\alpha }\frac{\left|∆x\right|}{R}=\;1-\frac{2}{\sin^{-1}(NA)}\frac{\left|∆x\right|}{R}$$

From the above relationships, *b'*/*a'* has a maximum value of 1 for *∆x* = 0, where the beam shape is circular. This ratio is also equal to one if the focal point is at any point on a flat surface sample. From Equation (3), *b'*/*a'* decreases linearly as a function of off-center displacement when the displacement is small compared to the radius of curvature of the object. It also indicates that a lens sample with a smaller radius of curvature will have a lower value of *b'*/*a'* for the same displacement.

Note that for the optical arrangement shown in Figure 1, when we move the lens sample to the left (in the −*x* direction), a segment of the circular beam image is removed from the left. When we move the lens sample to the right (in +*x* direction), a segment of the circular beam image is removed from the right.

We now consider the case where the probe’s focal point is moved at any displacement *r*(*∆x*, *∆y*) from the lens vertex, as shown in Figure 7e. We use Equation (2) to determine *b'*/*a'* for the beam shape, where the angle *∆*θ between ON_1_ and ON_2_ becomes:

(4) $$∆\text{θ}\;=\;\tan^{-1}\left(\frac{r}{\sqrt{R^{2}-r^{2}}}\right)\;≅\;\frac{\sqrt{∆x^{2}+∆y^{2}}}{R}\;;\;∆x\;\ll R$$

where $r=\sqrt{∆x^{2}+∆y^{2}}$. Note that the values of *a'* and *b'* are measured along the movement direction which has an angle of ϕ = tan^−1^ (*∆y*/*∆x*) (see Figure 7e).

The device sensitivity *S* for small displacement around the vertex can be determined from Equations (2) and (4) as:

(5) $$S\;≅\;-\frac{2}{R\;\sin^{-1}\left(NA\right)}\;;\;r\;\ll R$$

Therefore, if one needs a system with higher detection sensitivity, an objective lens with a lower NA must be used.

The measured *b'*/*a'* ratios of the reflected beam shape for a 2.5-mm-radius ball lens sample obtained from the beam images shown in Figure 3a and the corresponding calculated *b'*/*a'* ratios are given in Table 1 for comparison. The *b'*/*a'* ratio is calculated from Equation (2) by using the angle *∆*θ determined from Equation (4) given values of *∆x* and *∆y*. The calculated *b'*/*a'* ratios agree very well with the measured values, with an average difference of 2.7%. For larger displacements, significant differences between the experimental and the calculated values were observed. This is because our analysis was limited to small displacement around the lens vertex. These results indicate that our beam-shape analysis based on partial aperture blocking of the reflected beam by the aperture is indeed acceptable.

**Table 1:** Measured and calculated distorted radius ratios *b'*/*a'* of the reflected beam shapes for a 2.5-mm-radius ball lens sample for given values of off-center displacements *r*(*∆x*, *∆y*). Measured ratios were obtained from beam images shown in Figure 3a. The calculated ratios, in the parentheses, were calculated from Equations (2) and (4) with NA = 0.25 and *R* = 2.5 mm.

*b'/a'*		*∆x* (mm)						
		−0.15	−0.10	−0.05	0.00	0.05	0.10	0.15
*∆y* (mm)	−0.15	0.313	0.424	0.500	0.528	0.501	0.433	0.323
		(0.315)	(0.416)	(0.486)	(0.512)	(0.486)	(0.416)	(0.315)
	−0.10	0.405	0.546	0.641	0.682	0.655	0.551	0.415
		(0.416)	(0.539)	(0.634)	(0.672)	(0.634)	(0.539)	(0.416)
	−0.05	0.470	0.632	0.775	0.897	0.784	0.634	0.479
		(0.486)	(0.634)	(0.767)	(0.835)	(0.767)	(0.634)	(0.486)
	0.00	0.504	0.673	0.836	0.999	0.841	0.659	0.479
		(0.512)	(0.672)	(0.835)	(1.000)	(0.835)	(0.672)	(0.512)
	0.05	0.472	0.635	0.779	0.835	0.765	0.622	0.456
		(0.486)	(0.634)	(0.767)	(0.835)	(0.767)	(0.634)	(0.486)
	0.10	0.398	0.531	0.630	0.664	0.622	0.516	0.382
		(0.416)	(0.539)	(0.634)	(0.672)	(0.634)	(0.539)	(0.416)
	0.15	0.295	0.395	0.464	0.484	0.461	0.383	0.289
		(0.315)	(0.416)	(0.486)	(0.512)	(0.486)	(0.416)	(0.315)

## 5. Conclusions

We have demonstrated a fiber-optic confocal probe with an integrated camera that can be used to monitor the beam shape of reflected light. The integrated camera functions as a real-time vertex-sensing device. Placing the sensing spot at small displacements from the vertex point produces non-circular images that can be fitted to ellipses. We devised a calibration technique for the proposed system using a flat glass plate as a sample to obtain the reference circle and the reference origin. By analyzing the shape of the reflected light spot, we are able to precisely determine the location, both distance and direction, of the focal point of the confocal probe relative to the vertex of the measured lens. This device has a lateral resolution 1.2 µm for a 2.5-mm-radius ball lens. Beam-shape parameters obtained from analysis based on partial aperture blocking are in agreement with experimental data for small off-center displacement. This proposed confocal probe is suitable for high-precision central thickness measurement of small ball lenses or other optical elements, at a much lower cost than a three-dimensional surface profilometer.
