# Erratum: Sutapun, B. *et al*. Development and Beam-Shape Analysis of an Integrated Fiber-Optic Confocal Probe for High-Precision Central Thickness Measurement of Small-Radius Lenses. *Sensors*, 2015, *15*, 8512-8526

**DOI:** 10.3390/s16050698

**Published:** 2016-05-14

**Authors:** Boonsong Sutapun, Armote Somboonkaew, Ratthasart Amarit, Sataporn Chanhorm

**Affiliations:** 1 School of Electronic Engineering, Institute of Engineering, Suranaree University of Technology, 111 University Ave., Muang, Nakhon Ratchasima 30000, Thailand; boonsong@sut.ac.th; 2 Photonics Technology Laboratory, National Electronics and Computer Technology Center (NECTEC), National Science and Technology Development Agency (NSTDA), 112 Thailand Science Park, Pahol Yothin Rd., Klong Luang, Pathumthani 12120, Thailand; armote.somboonkaew@nectec.or.th (A.S.); ratthasart.amarit@nectec.or.th (R.A.); sataporn.chanhorm@nectec.or.th (S.C.)

The source of the funding of this work disclosed on the published paper [1] was incorrect. The authors would like to correct the acknowledgements of this article as follows:

**Acknowledgments:** This work was funded by Suranaree University of Technology’s research and development fund. 

The authors would like to apologize for any inconvenience this may have caused to the readers.
